# Psychological Tests or Rubbish?

**Published:** 1918-03-30

**Authors:** 


### The Hospital

The Workers' Newspaper of Administrative Medicine and Institutional Life, Administration, National Insurance and Health.

No. 1660, Vol. LXIII. SATURDAY, MARCH 30, 1918. PRICE ONE PENNY

### PSYCHOLOGICAL TESTS OR RUBBISH?

According to the *Scientific American*—the average American is nothing if not scientific—the recruits to the United States Army are put through a series of psychological tests in order to determine their "mentality"—blessed word—before they are incorporated in the regiment. The tests are supposed to settle, in the first place, whether they are of such "low mentality" (it would not be scientific, we suppose, to say stupid), that they cannot be allowed in the army at all; and in the second place, what kind of position or work in the army they are best fitted for. The men are tested a hundred or so at a time, by a series of tests of gradually increasing difficulty. The first thing required of the candidates—we beg pardon, the recruits—is to make a mark in the largest square in a row of squares. Then tests of the same kind but increasing in difficulty are set. Next they are tested for memory. The examiner reads aloud a set of three figures, and at the word "Go," the candidates—recruits—write them down. The number of figures in the set is gradually increased, and the number of recruits who drop out of the running increases *pari passu*. Then a set of mingled words is given, and is to be arranged in a sentence and so forth, and so on, and from the way in which the candidates—it is no use, we must call them candidates, for they are candidates—from the place they attain in the examination, a decision is come to as to whether they are fit for the charge of a squad, or for sentry duty; or to dig trenches, or only to peel potatoes. The results are said to form a valuable basis for promotion, or for the assignment of men for special duties.

It seems a pity that the authorities of the United States Army, while they are about it, do not apply the system throughout, make it thoroughgoing, and apportion a man's rank in the army by the results of a psychological examination. Thus, if a man can remember and write down no more than two figures he is only fit to peel potatoes. If he can remember three, he goes into the ranks as full private. Ability to remember four figures and write them down correctly should qualify him to be a corporal; five, a sergeant; six, a sergeant-major; seven, a subaltern, and so on until we reach the Field-Marshal C.I.C., who should be able to memorise at least four-and-twenty figures. How easy it all is, and what a Commander-in-Chief the man of twenty-four figures would make! In our unscientific English way we look to a man's record, and guess, more or less inaccurately, what he is likely to do in the future from what he has done in the past. The man who has done best as private is made a corporal, the man who has done best as corporal is made a sergeant; and the Commander-in-Chief is chosen from those subordinate commanders who have shown most ability in subordinate but quasi-independent commands. It is evident that this system offers abundant opportunity for favouritism, nepotism, and illegitimate influences of every kind, while the figure test would be quite unassailable on this score, at any rate.

Frankly, it is all rubbish. "Psychology" is a word to conjure with, and "Psychological tests" has a fearsome sound, and seems to indicate something profound, something exact, precise, unimpugnable—in fact something extremely and eminently Scientific, with a very capital S. It is all rubbish. The way to find out whether a man can peel potatoes is not to set him to cross out squares or answer conundrums, or remember figures, but to show him how to peel potatoes and then see how he does it. And so it is with digging trenches, leading a squad, or commanding an army in the field. There is only one way worth a straw of determining whether or not a man can do a thing, and that is by setting him to do it, and observing how he does it, and what the result is when he is done. The same foolish mistake has been made in testing children as to whether or not they are so feeble-minded as to be unable to earn their living. They have been put through a series of tests in the "psychological laboratory"; they have been put through a course of Experimental Psychology, which is as much as to say they have been set to play with very expensive toys, which make marks on diagrams and draw lines on charts. But people do not—except Experimental Psychologists—make their living by playing with expensive toys. They make their living by doing work that has a market value; and the only way to discover whether they can do such work is to set them to try to do it, and when they have tried, to take their work to market and find out what it will fetch. That is the way and the only way. Many an idiot could beat Lord Furness or Lord Northcliffe at remembering figures and doing sums; but in spite of their inferiority in remembering figures and doing sums, Lord Furness and Lord Northcliffe have continued to earn their respective livings, and perhaps two millions over; while the calculating idiot, who could knock them into a cocked hat at sums, has never made a farthing in his life, and never will. Experimental Psychology is an unholy fraud.

